# Biologically encoded magnonics

**DOI:** 10.1038/s41467-019-12219-0

**Published:** 2019-09-25

**Authors:** Benjamin W. Zingsem, Thomas Feggeler, Alexandra Terwey, Sara Ghaisari, Detlef Spoddig, Damien Faivre, Ralf Meckenstock, Michael Farle, Michael Winklhofer

**Affiliations:** 1 Faculty of Physics and Center for Nanointegration (CENIDE), University Duisburg-Essen, 47057 Duisburg, Germany; 2 Ernst Ruska Centre for Microscopy and Spectroscopy with Electrons and Peter Grünberg Institute, Forschungszentrum Jülich GmbH, 52425 Jülich, Germany; 3 Department of Biomaterials, Max-Planck Institute of Colloids and Interface Science, Golm, 14476 Potsdam, Germany; 4 CEA/CNRS/Aix-Marseille Université, UMR7265 Institut de biosciences et biotechnologies, Laboratoire de Bioénergétique Cellulaire, 13108 Saint Paul lez Durance, France; 5 Institut für Biologie und Umweltwissenschaften, Carl von Ossietzky Universität Oldenburg, 26129 Oldenburg, Germany; 6 Research Center Neurosensory Science, University of Oldenburg, D-26111 Oldenburg, Germany

**Keywords:** Applied microbiology, Biomineralization, Magnetic devices

## Abstract

Spin wave logic circuits using quantum oscillations of spins (magnons) as carriers of information have been proposed for next generation computing with reduced energy demands and the benefit of easy parallelization. Current realizations of magnonic devices have micrometer sized patterns. Here we demonstrate the feasibility of biogenic nanoparticle chains as the first step to truly nanoscale magnonics at room temperature. Our measurements on magnetosome chains (ca 12 magnetite crystals with 35 nm particle size each), combined with micromagnetic simulations, show that the topology of the magnon bands, namely anisotropy, band deformation, and band gaps are determined by local arrangement and orientation of particles, which in turn depends on the genotype of the bacteria. Our biomagnonic approach offers the exciting prospect of genetically engineering magnonic quantum states in nanoconfined geometries. By connecting mutants of magnetotactic bacteria with different arrangements of magnetite crystals, novel architectures for magnonic computing may be (self-) assembled.

## Abstract (web summary)

The capability to engineer magnon states in confined geometries is vital to future nano-magnonics. Here the authors demonstrate that the topology of the magnon bands is determined by the local arrangement and orientation of nanoparticles and can be controlled by the genotype of magnetotactic bacteria.

## Introduction

Future demands for computational power cannot be met by the current electrically powered silicon-based technology due to fundamental physical and economical limits in power consumption, generation of heat, and electromigration^1,2^. While the computing capacity of the human brain is equivalent to exaFLOPS (10^18^ floating point operations per second)^3^, operating at a moderate 37 °C, the most advanced super computers today are limited to petaFLOPS (10^15^) whilst being immensely power-hungry in order to perform such computations and even more so to dissipate the heat which is generated in the process. This problem has led  to the investigation of alternative approaches to integrated micro processing like quantum or magnonic computing^4–12^. Other future concepts envision parallel computation networks inspired by macromolecular chemistry and cell biology, exploiting the possibilities of chemically controlled self-assembly—for example cytoskeletal filaments propelled by motor proteins through a hierarchical mesh of nanofabricated channels^13^.

Here we suggest the combination of biology and solid state magnetism to control magnetic quantum excitations—called magnons—in genetically engineered networks of magnetotactic bacteria for resource efficient computing. We demonstrate that spin wave dispersions can be modified through bacterial genetics, paving the way towards bio-magnonic computing. Although such magnetic nano-assemblies can be constructed in various ways, this biogenic approach yields the potential to drastically reduce the environmental footprint^14^ of electronic devices, as biological cells are used instead, and the active material (magnetite, Fe_3_O_4_) is sustainably recyclable. Furthermore, the use of self-reproducing biological cells allows for a low cost natural exponential upscaling. Additionally, magnons as carriers of information drastically reduce energy demands as compared to conventional electronics because less energy is needed to excite a magnon and negligible amounts of energy are dissipated into heat^8,12^. Another advantage of magnonic logic gates is the prospect of using operation frequencies in the terahertz regime^15^, three orders of magnitude above what is achievable in current semiconductor electronics. Thus, a magnon processor the size of a conventional CPU chip would not only beat current silicon technology (Supplementary Fig. 1), but has the potential to outperform the human brain, scaling the ambitious computing facility envisioned by the Human Brain Project^3^ down to a single chip—as is demonstrated in the supplementary text.

Magnonic devices based on lithography have been considered for many innovative, energy efficient computer technologies such as information transport and logic circuits operating at high efficiency^8,16^, but thus far have been realized on the micrometer scale only^4–7,10,11^. We demonstrate the potential of nano-sized biogenic magnetite crystals as truly nanoscale magnonic devices using bacteria with genetically encoded arrangements of nanoscale, dipolar-coupled magnets. This biomagnonic approach offers the perspective of tailoring future computing devices, employing biological tools such as directed evolution^17^, genetic engineering, or synthetic biology^18^.

Spin waves—or their quanta called magnons—are collective oscillations of dipolar or exchange-coupled magnetic moments in a magnetic solid. They can be excited thermally or via microwaves at energies as low as 10 µeV. Their wavelength can be substantially shorter than the corresponding wavelength of electromagnetic waves in vacuum and controlled by geometry and size (finite size effects and nanoscale geometry) as well as by magnetostatic coupling between magnetic moments. Magnonic information exchange is mediated by Joule-heat-free transfer of spin information over long distances, which makes spinwaves excellent candidates for so-called magnon spintronics and computing^4–12^.

In the following, we experimentally show and computationally confirm that the magnonic fine structure of nanoparticle chains, and thereby the spatial amplitude and phase profile, can be altered by means of genetic mutations in magnetotactic bacteria. As an experimental realization of a nano-magnonic system, we analyzed magnetosome chains in the magnetotactic bacterium *Magnetospirillium gryphiswaldense* (strain MSR-1, wildtype), whose well-defined magnetic properties have been comprehensively studied before^19^. These chains are composed of magnetite single crystals, which are magnetic single domains and oriented with one of their magnetic easy <111> axes along the chain axis^20–24^. We selected cells containing a single-strand chain each, consisting of up to 12 magnetite crystals (35 nm crystal size) arranged in a linear configuration within the cell body. Each crystal is enclosed in a vesicle composed of a lipid-bilayer membrane (ca. 4 nm thickness)^25^, and different membrane proteins controlling the magnetosome chain formation^26^. The organic, non-magnetic, spacer material separates adjacent magnetic crystals by at least 8 nm^25^ and thus completely suppresses magnetic exchange interactions between them. Hence, collective magnetic phenomena in the magnetosome chain, such as spinwaves, are purely mediated by magnetostatic coupling between the particles.

## Results

### FMR spectra of a single magnetosome chain exhibits magnonic band gaps

**Fig. 1:**
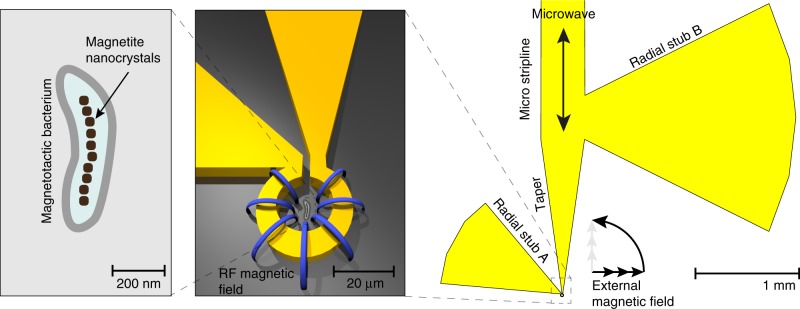
Measurement principle: The magnetobacterium cell (left) is positioned inside the omega-shaped cavity of a microresonator (see also Supplementary Fig. S2) and is exposed to an RF-magnetic field (blue field lines) produced by the microwave electric current. The microwave energy, which is coupled into the micro stripline is tuned to the eigenfrequency of the microresonator, geometrically defined by stubs A and B. A static external magnetic field is applied in the plane of the resonator setup and alters the resonance frequency of the particles as the field strength is decreased (Eq. 1). If one or multiple particles resonantly absorb at the fixed microwave frequency, the eigenfrequency of the system is perturbed, resulting in an increase of the reflected microwave power (a spectral peak). Each spectrum is acquired by sweeping the magnitude of the external magnetic field at a different angle

**Fig. 2:**
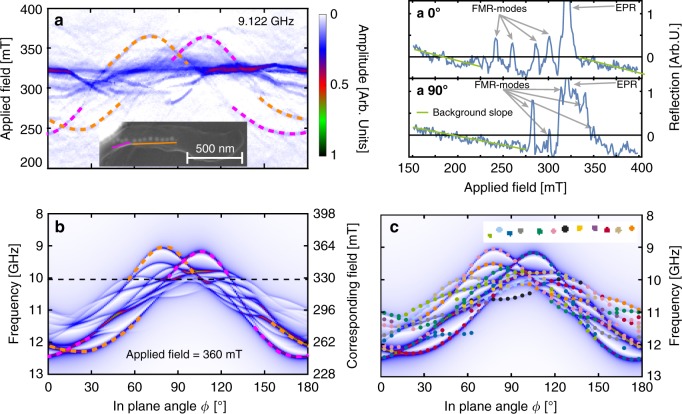
Micro-FMR spectra of a single magnetic bacterium containing a straight chain of about a dozen magnetic nanocrystals. **a** Angular dependence of FMR spectra for the cell shown in the inset with magnetite particles appearing as bright dots; for each of the 180 angles, an individual FMR spectrum was recorded from 150 mT to 400 mT magnetic field strength. Two single spectra recorded at 0° and 90° are shown to the right (**a 0°**) & **a 90°**)). The amplitude is color-coded according to the color scale next to a), which is used throughout. The high-amplitude band at about 320 mT (*g* = 2.0 at 9.0 GHz) corresponds to an EPR (electron paramagnetic resonance) background. The two main FMR lines (marked with dashes) have maxima at 70° (355 mT) and 110° (355 mT), respectively, and are caused by the two main segments of the chain. (Inset) SEM micrograph of the magnetotactic bacterium (MSR-1, wildtype) in the resonator. The left, short segment of the particle chain is offset from the main chain segment. The bright contrast band on the top left a section of the resonator loop. **b** FMR spectra obtained from a micromagnetic model of the magnetosome chain, computed for an applied field of 360 mT in the frequency domain (which is reciprocal to the field domain according to Eq. 1). As a guide to the eye, the two prominent lines corresponding to the anisotropy of chain segments are highlighted in accordance with **a**. The black dashed line indicates the position where the EPR line would be. **c** Each resonance line is marked in the color of the particle (inset) that gives the major contribution to that line, to help understand where a resonance mode is localized in the chain

To study magnetotactic bacteria at the single cell level we used a resonant microcavity (ref. ^27^, see Fig. 1 and Supplementary Fig. 2) at X-band microwave frequency (9.1 GHz) to excite and detect dipolar coupled (magnetostatic) spin waves in the magnetosome chain as a function of the strength and in-plane angle of a homogeneous magnetic field at room temperature. In the resulting ferromagnetic resonance (FMR) absorption spectra (Fig. 2a), one can recognize several FMR lines characterized by a strong angular-dependent anisotropy. The spectra exhibit a 180° periodicity, and the two most prominent lines (dashed) reveal the prevailing uniaxial shape anisotropy of the two linear chain segments, as confirmed with a micromagnetic model (Fig. 2b). For both lines, the anisotropy splitting ranges from 240 mT (the so-called low-field resonance) to 355 mT (high-field resonance), which indicate magnetostatically easy and hard axes, parallel and perpendicular to a chain axis, respectively. As can be seen from individual spectra in (Fig. 2a 0°) and (Fig. 2a 90°), the typical resonance line-width is only about 1 mT, much narrower than the ca 40 mT—broad lines observed in a magnetically pre-aligned sample containing billions of magnetotactic bacteria cells^28^. Previous integral FMR absorption spectra of such bulk samples of cells^19,28–30^ were explained by a theoretical model assuming that each chain responds like a single particle (ellipsoid) with an effective uniaxial anisotropy along a cubic <111> axis^31^. Here we show that the experimental FMR spectrum of a single cell (Fig. 2a) exhibits a diversity of features that are far beyond the simplified single particle model. Most notably, we observe a formation of spectral splitting of resonance modes in their angular dependence. Due to the strong exchange coupling inside each particle, continuous spatial variations of the phase within a given particle are forbidden (i.e., nonzero k-vectors cannot be accommodated within a particle). Thus, spectral gaps between different mode patterns are, in fact, band gaps in the k-dependent spin-wave spectrum. This peculiarity can, for example, be observed at 90° (between 270 mT and 300 mT), which lies between the hard directions of the two major chain segments. Further examples of band-gaps can be seen throughout the spectrum. Another interesting aspect are bands that have unusual curvature, with the most pronounced example being the flat band ranging from 45° to 90° at 290 mT.

### Micromagnetic understanding of FMR spectra

To gain insight into the origin of the evolution of these resonances, we performed GPU-based micromagnetic calculations using MuMax3^32^. We adopted the geometry of the model structure from the scanning electron microscopy (SEM) in-plane projected images of the magnetosome chains and used the material parameters for magnetite (see “Methods”). To model the microwave excitation, we designed a spatially uniform, time-dependent field pulse containing all frequencies between 1 and 29 GHz with equal amplitude. The dynamic magnetic response was obtained in the frequency domain by Fourier transformation. This procedure was performed for two applied magnetic field strengths in saturation to confirm that all frequency-dependent features project linearly under variation of the applied field, i.e.,

1 $$f = \frac{{g\mu _{\mathrm{B}}}}{h}\left( {B_{{\mathrm{app}}} + B_{{\mathrm{anis}}}} \right)$$

where *g* is the g-factor (2.1 for magnetite^33^), *μ*_B_ is the Bohr magneton, *h* is the Planck-constant, *B*_app_ is the applied field, and *B*_anis_ is the anisotropy field, which here, i.e., for sufficiently large applied fields, does not depend on the applied field^34^. We observe a good agreement between experimental (Fig. 2a) and simulated spectrum (Fig. 2b), in terms of the resonance field range, band gaps, and band deformations. Based on the micromagnetic analysis, we can identify the origin of the two main modes in the experimental spectrum (Fig. 2). The majority criterion applied to the data in Fig. 2c allows a more detailed view on which mode is dominated by which particle. For example, modes dominated by the light green and orange particle at either chain-end exhibit much smaller anisotropy than modes dominated by the pink particle. Their anisotropy span (Δf) is about 1.5 GHz, while the pink particle dominates modes spanning ca. 3 GHz. This reduction in effective anisotropy occurs because these end particles have only one neighbor each, and therefore experience smaller local effective dipolar-induced anisotropy, hence a lower *B*_anis_. Remarkably, continuous lines are not always dominated by the same particle. For example, the pink particle dominates the intensive mode at low field (260 mT, 0°) until about 30°, where the dark green particle takes over. In the angular range between 30° and 55°, the pink particle jumps across a spectral gap and dominates a different mode at high field from 50° onward; from 140° to 160° the pink particle has a large part in two different resonance modes. One between 10 and 11 GHz, the other one between 11 and 12 GHz. Similar features can be observed throughout the spectrum.

**Fig. 3:**
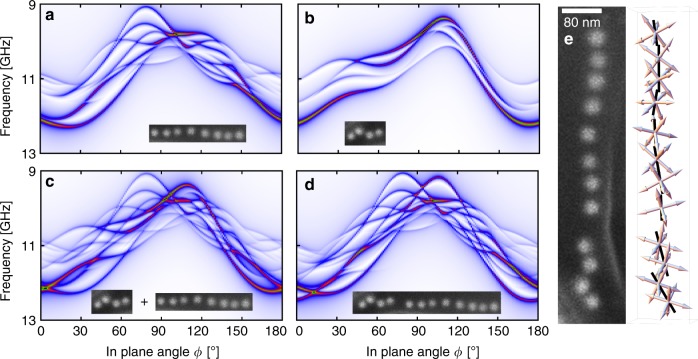
Micromagnetic dissection of a magnetosome chain. Simulated FMR spectra for different segments of the magnetosome chain shown in inset of Fig. 2a. **a** Long segment with eight particles in a roughly linear configuration. **b** Short segment with the four particles which are slightly offset from the main segment. **c** The superposition of the spectra (**a**) and (**b**). **d** Complete chain. The difference between the spectra (**c**) and (**d**) is due to magnetostatic interactions between the two chain segments present in the twelve-particle model (**d**). **e** Left, enlarged SEM micrograph of the particle chain from the inset in Fig. 2a. Right, geometric model of the particle chain, showing the <100> crystallographic axis system (arrows) at the center positions of each particle (as determined from the SEM micrograph). The crystallographic axis system of each particle is assumed to be oriented such that one of its four <111> magnetic easy axes, shown as black rod, is aligned towards the nearest neighbor particle. The spatial orientation of the other three <111> axes of the particle varies randomly from one particle to the next. In magnetite, at temperatures above the isotropic point of 135 K, the <100> axes represent magnetic hard axes and the <111> axes represent magnetic easy axes

To further understand the emergence of spectral features, we have separately modeled the two segments of the chain consisting of eight and four particles, respectively (see Fig. 3). The spectra shown in Fig. 3a, b confirm the assignment of the experimental spectrum (Fig. 2a) to the chain segments. More importantly, the simulated spectrum of the complete chain (Fig. 3d) has fewer lines compared to the superposition (Fig. 3c) of spectra a and b. This reduction is a direct result of coupling between the two segments, which promotes collective oscillations. Another consequence of the inter-segment coupling is an increase in the anisotropy in the short chain segment by about 0.5 GHz.

### Geometry of magnetosome chain determines magnonic band structure

**Fig. 4:**
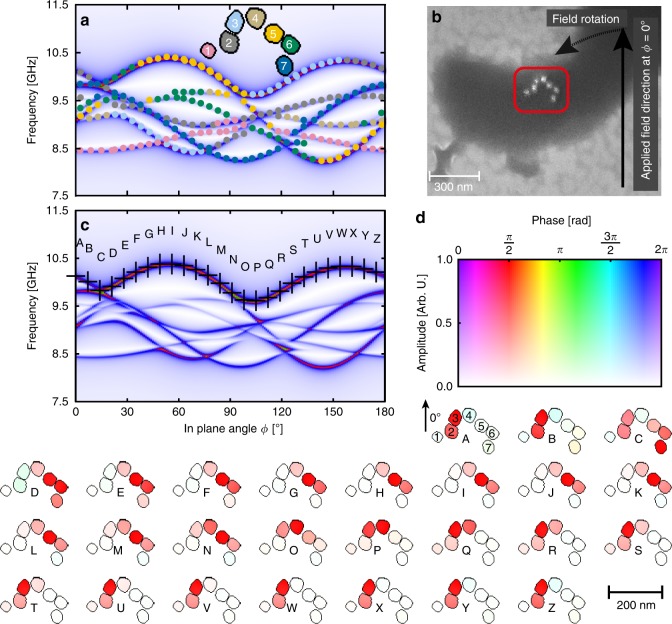
Correlation of magnonic fine structure and geometric configuration of particles. **a** Simulated FMR spectra of seven magnetic nanocrystals in a coiled arrangement (see inset) reproduced as silhouettes from an electron micrograph (b) of a cell of the ∆mamK-mutant of MSR-1 (experimental spectra see Supplementary Fig. 4b). In (**a**), each resonance line is marked in the color of the particle that gives the major contribution to the amplitude of that line. The two end particles in (**a**) (blue and pink) are weakly coupled to the chain and their resonances are unperturbed over most of the angular range. Their uniaxial sin(2ϕ)-dependence, however, become distorted or even interrupted where a resonance line of the nearest neighbor is encountered. E.g., at about 100°, the pink particle most strongly interacts with the gray particle because the applied field is coaxial with the connecting line between the two particles. In consequence, the pink mode is deflected toward the gray mode with which it merges. In contrast, at 120°, the external field destabilizes the interaction between the orange particle and the green particle, so that the green mode can interact with the blue mode, causing a band gap in the blue mode. The apparent sin(4ϕ) dependence of the pronounced resonance line on the top is a sequential merger of the five in-chain particles resonating one after another as the field angle is varied around the chain bend. Thus, each particle’s resonance field/frequency mostly follows its local effective anisotropy. **c** Analysis of the spatial contributions to the upper-envelope line of the spectrum. Each letter corresponds to a position on the envelope and the phase and amplitude of the magnonic response is mapped according to the color scale (**d**) onto each point of the simulation grid (2.6 nm mesh size). Phase and amplitude are uniform within a given particle, with red hue indicating resonant response (90° phase relative to excitation) and cyan hue indicating opposite-to-resonant response. The standing spin wave associated with this continuous resonance line (envelope) shifts through the discrete elements of the chain as the magnetic field angle is varied

Since the nonlinearity of the chain structure in Fig. 2 appears to be responsible for a multitude of features, we next selected cells containing strongly curved magnetosome chains of the so-called Δ*mamK* mutant of the bacterium (Fig. 4b and Supplementary Fig. 3). The mutant lacks MamK, an actin-like cytoskeleton protein, which in the wildtype has a role in positioning the magnetosome chain in a midcell location and in equipartitioning it among daughter cells^35–37^. As a result of the lack of this protein, we observe an increased amount of strongly curved and dendritic particle assemblies (Supplementary Fig. 4). Because of the chain curvature, the experimental spectrum (Supplementary Fig. 3c) of just two cells of the Δ*mamK* mutant exhibits a much broader variety of resonance lines, each following a different angular dependence. To gain microscopic insight into the correlation of spectral fine structure and geometric arrangement, we fed the SEM image (Fig. 4b) into our micromagnetic model. As we learned from the simulations of the straight chain (Figs. 2, 3), the dominant anisotropy is due to particle configuration (local effective shape anisotropy). Therefore, we now use the simplest model capable of explaining the essential features of the measured spectrum (i.e., band gaps and band deflection). That is, we here neglect higher order energy density terms such as cubic magnetocrystalline anisotropy, which is one order of magnitude smaller than the magnetostatic energy in the particles with nearly cubic shape.

Analogous to Fig. 2c, we applied a majority criterion to the simulated spectra to understand the microscopic origin of the band structure. From this representation (Fig. 4a), one can see that the continuous resonance line at the top (upper envelope) originates from chain segments that are approximately aligned with the external field, in the sense that the line is composed of easy axis resonances; similarly, the lower boundary of the magnon diagram is composed of hard axis resonances only. For example, the upper envelope is dominated by particle 5 between 40 and 100°, because this chain segment experiences the largest projection of the external magnetic field compared to the other segments. At 100°, the amplitude shifts around the bend of the chain, and particle 3 dominates from 100° to 150°, until particle 2 takes over. Thus, the absorption amplitude moves through the chain from particle to particle as a function of the field angle and causes magnon band deformation.

**Fig. 5:**
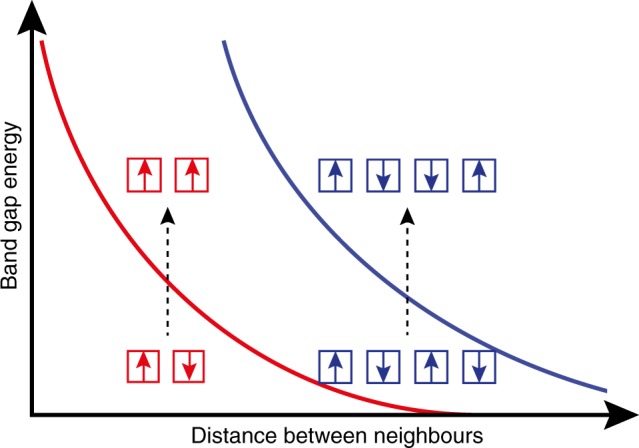
Relation between energy gap and spatial gap. The energy gap (solid lines) between magnonic eigenstates (modes) of coupled particles decreases with the particle distance and depends on the number of particles involved in the modes. The arrows indicate the phase of the oscillating magnetic moment in each particle (squares). The red curve shows the band gap between the antiparallel oscillation mode and the parallel oscillation mode, i.e., same phase versus opposite phase resonance. The analog in a continuous system would be the transition between a uniform mode and a mode where the wavelength is equal to the length of the system. The blue curve shows the band gap between an antisymmetric short-wavelength mode (lower blue squares) and a symmetric mode with twice the wavelength (upper blue squares). Closely spaced particles (which have stronger dipolar coupling) exhibit larger spectral gaps between such modes than distant particles do. The discontinuous nature of the particle chain discretizes the modes in k-space, such that these spectral gaps in the collective eigenmode spectrum are also band gaps

At the same time, each continuous line segment tracks the local effective anisotropy of one or multiple particles, that is, particles inside shorter chain segments have smaller anisotropies than particles in longer chain segments, for example, compare the pink (1) and yellow (5) line in Fig. 4a. However, each particle can only react to its local dipolar-induced anisotropy where band deformation is not favored, thus creating band gaps. In other words, band gaps are induced by dipolar coupling between neighboring particles i.e. closely spaced particles induce larger band gaps where the resonance lines cross, whilst particles that are further apart cause smaller or no band gaps. This principle can be generalized to correlate the experimentally observed spectral gaps with spatial gaps, as schematically illustrated in Fig. 5.

Furthermore, band gaps can be observed for more complex modes involving multiple particles, which can accommodate different standing spinwave patterns. These are dipolar coupled modes in which each particle resonates uniformly, albeit with different phase relationships between the particles. Examples for simple band gaps can be seen in Fig. 4a near 30°, where the resonances of particle 2 (gray) and 3 (light blue) show repulsive behavior, resulting in a small bandgap. Similarly, at about 100°, a splitting of resonances between particles 1 (pink) and 2 (gray) occurs. As expected, the splitting is smaller for resonances involving next-nearest neighbors than for nearest neighbors. Consequently, no band gap is observable near 65°, where the end particles 1 (pink) and 7 (dark blue) resonate.

### Spatial localization of spin-wave amplitude

Building on the knowledge that the geometric chain features have a direct influence on the magnonic structure, we next analyzed the spatial distribution of magnon amplitude to understand the microscopic origin of the angular-dependent band structure. For this purpose, we present in Fig. 4c, the spatial distribution of spin-wave amplitude exemplarily for the continuous resonance line forming the upper envelope of the FMR spectrum simulated for the curved chain. It now becomes apparent that the envelope line does not represent the anisotropy of a single chain segment, but, surprisingly, represents a standing spin wave that shifts through the chain as the magnetic field angle is changed. Thereby, the spin-wave amplitude is always localized in those chain segments that are approximately aligned with the external field, in the sense that the line is composed of easy axis resonances, as was already inferred from the majority criterion (Fig. 4a). At 150° (position V, W in Fig. 4c), the amplitude is localized in particle pair 2 and 3, then at 100° (O-Q) in pair 3 and 4, and at 60° (H-J) in the triplet 4, 5, and 6.

As a general rule, we observe that *coupled* systems avoid line crossings and instead exhibit band deflection at points where the resonances of *decoupled* systems would otherwise cross, which can be most prominently observed at O-R in Fig. 4c. This effect leads to unusual line deformations, which deviate from the symmetry of chain segments and are mediated by a spatially shifting spin-wave amplitude. On the other hand, when looking at any short enough line segment, one finds the local effective anisotropy of one or multiple particles to be represented. For example, the triplet 4, 5, 6 in Fig. 4c has a resonance line tracking its anisotropy along the path from F to M. This tracking is further illustrated in Fig. 4a, where the local effective anisotropy of individual particles becomes apparent although the lines are intersected by band gaps.

## Discussion

In summary, our results suggest the first step to a novel nanomagnonic concept based on dipolar coupled assemblies of nanoparticles. We further illustrate that there is leverage to genetically engineer and biologically grow such nanoparticle ensembles suggesting a new research toward biomagnonics. In our room temperature nanoscale magnonic device, i.e., a magnetosome chain, each particle performs spatially uniform oscillations because the particle size is well below the wavelength of so-called exchange spin waves^38^. As opposed to a diluted magnetic nanoparticle system, where each particle’s resonance mode represents a continuous function of the applied field angle, the proximity of the particles in a dipolar coupled assembly promotes complex phase relationships and thus produces distinct features such as band gaps and band deformations. These features can be harnessed for device applications as they are tunable via the geometric arrangement and morphology of the particles. The underlying biological machinery used in this work may in future be exploited by genetic manipulation and directed evolution toward magnonic devices with desired transport properties.

## Methods

### Cultivation of magnetotactic bacteria

*M. gryphiswaldense* strain MSR-1 (DSM6361) as well as Δ*mamK* mutants were used in this study. The Δ*mamK* strain (ref. ^35^) was a gift from Dirk Schüler at University of Bayreuth. The cultivation medium was prepared as described earlier^39^. Briefly, 20 mL of cells from a subculture were transferred to 200 mL of medium in 0.5 L rubber-sealed flasks, with microaerobic conditions (1% O_2_ in the headspace). Then, the flasks were incubated under gentle shaking (100 Rpm) at 28°C for 24 h (72 h for Δ*mamK*).

### Sample preparation

Bacteria cells were mixed with HEPES (2-[4-(2-hydroxyethyl)piperazin-1-yl]ethanesulfonic acid) buffer at pH = 7 to prevent them from osmotic bursting. The mixture of bacteria, nutrient medium, and HEPES was then centrifuged at 9000 rpm for 5 min at a temperature of 4 °C. Four centrifuge runs were performed and, after each run, the supernatant was substituted with HEPES buffer. The resulting pellet containing the bacteria cells then was re-dispersed in buffer solution, diluted, and transferred into a microcapillary mounted on a micromanipulator unit to deposit single cells of bacteria in a microresonator (Fig. S2). To obtain the single-cell sample shown in Figs. 2a, 3e, we used a focussed-Ga-ion beam (FEI Helios NanoLab^TM^ 600 Dual beam FIB/SEM system) to ablate extra cells of magnetic bacteria in the microresonator; electron micrographs from the samples (Figs. 2a, 3e and 4b) were obtained with the secondary electron detector in the FIB/SEM system at 10 kV acceleration voltage. After SEM inspection, the microresonator was connected via a low-loss semirigid coaxial cable to a conventional microwave bridge (Varian E102). The spectra were acquired with a modulation field of 0.5 mT and 123.45 Hz frequency and recorded with a Stanford-Research SR 830 DSP lock-in amplifier (original recordings available as SI material).

For transmission electron microscopy (Supplementary Fig. 4), cells from a pellet were dropped on a grid, air dried, and imaged in bright-field mode at 120 kV under an EM 912 Omega (Carl Zeiss Oberkochen).

### Micromagnetic simulations

Simulations were set up in mumax3^32^ version 3.9.1 for the straight chain shown in Figs. 2 and 3 and for each of the curved chains shown in Fig. 4 and Supplementary Fig. 3 individually. Rather than using idealized geometries with perfectly aligned particle faces, which may produce symmetry-induced artifacts, we approximated the lateral morphology of a chain by binarizing SEM images. The binarized images where rescaled such that each pixel in the image corresponds to a pixel in the simulation grid. The height of each particle was fixed to a value of 36 nm. The cell size was set to 2.6 nm by 2.6 nm by 8 nm with a grid size of 64 by 128 by 6 elements. The simulation parameters, chosen to approximate magnetite, where set to Aex = 1.32e−11 [J/m]^40^, Msat = 4.8e5 [A/m], k1c = −1.10e4 [J/m^3^]^33^, and gammaLL = 1.85556e11 [rad/(Ts)] corresponding to a g-factor of 2.11^33^. For the simulations of the linear chain with kink (Figs. 2 and 3) we included the cubic magnetocrystalline anisotropy. For the simulations of the crooked and ring-shaped chains (e.g., Fig. 4 and Supplementary Fig. 3), we use the simplest model capable of explaining the essential features of the measured spectrum (i.e., band gaps and band deflection). That is, we here neglect cubic magnetocrystalline anisotropy, which is one-order of magnitude smaller than the magnetostatic energy in the particles with nearly cubic shape.

The following procedure was performed for each chain separately for static field magnitudes of 280 and 360 mT. The applied field angle was iterated from 0° to 180° in steps of 1° in the plane of the chains. For each applied field configuration the magnetization of the system was allowed to relax into energetic equilibrium. Then a time dependent field pulse was modeled as a sinc function containing all frequencies from 0 to 30 GHz and the simulation was ran such that each frequency contributes with approximately the same amplitude. An example mumax input file for a chain consisting of eight particles (whose full angular dependence is shown in Fig. 3a) is available as Supplementary Information.

## Supplementary information

Supplementary Information

Peer Review

Source Data

## Data Availability

The Supplementary Information contains the measured FMR data from Fig. 2a in tab-separated text format as well as an exemplary MuMax input script for the micromagnetic simulation of a magnetosome chain exposed to a broadband pulsed magnetic field. All relevant simulation output data are available from the authors upon request.

## Code availability

Our custom Java program for extracting the dynamic response of each cell of the simulated micromagnetic structure is available at: https://github.com/BenjaminWZingsem/Spatial_FFT_MumaxData.git along with a series of MuMax output files that we simulated and processed for the paper (Fig. 3a) is available as Supplementary Information.
