# Supplementary material for: Biologically encoded magnonics
Source: Nat Commun. 2019 Sep 25;10:4345. doi: 10.1038/s41467-019-12219-0 (PMC6761176; doi:10.1038/s41467-019-12219-0)
Supplement: Supplementary file 1 — Supplementary Information [file 41467_2019_12219_MOESM1_ESM.pdf]

Biologically Encoded Magnonics

B. W. Zingsem, T. Feggeler, A. Terwey, S. Ghaisari, D. Spoddig, D. Faivre, R. Meckenstock, M. Farle & M. Winklhofer

SUPPLMENTARY SECTION

SUPPLEMENTARY NOTE 1:

ESTIMATION OF TRANSISTOR COUNT

Consider a conventional, 8 core intel core i7 processor. The transistor count is approximately $2.6 \cdot 10^9$. The size of the processor package is about 40 mm by 40 mm with a thickness of the order of 5 mm [source: https://www.intel.com/content/dam/www/public/us/en/documents/guides/core-i7-lga2011-3-tmsdg.pdf], depending on the socket. Considering 14 nm lithography, the typical transistor size is of the order of 100 nm by 100 nm depending on what is considered to be part of an individual transistor. For example, with an area transistor density of 100% across the die area, the i7 6700T with its 122 mm^2 die and its $1.35 \cdot 10^9$ transistors, an effective transistor size can be estimated to 300 nm by 300 nm. Being a gross over estimation due to the negligence of interconnections and die boundaries a better estimation should only consider the lithographically patterned part of the die, which is about 25% to 50% of the die size. This results in an effective transistor size of about $\sqrt{0.25}$ to $\sqrt{0.5}$ times 300 nm, or about 150 nm by 150 nm to 210 nm by 210 nm. Still neglecting in-plane connections, this remains an over estimation of the transistor size by up to 30% and shall only serve as a figure of merit. A typical magnon logic gate as presented here would have a size of about 100 nm by 100 nm. A magnon processor, similar in size to the lithographically patterned area of a CPU die, would thus have a comparable transistor count. However, the transistor count could in principle be much larger in plane, since heat is not an issue. Additionally, the third dimension is open to be fully occupied with magnonic gates as they no longer require to be near a heat sink. The number of magnon gates in a volume of $122\ \mathrm{mm}^2$ by 5 mm = $6.1 \cdot 10^{-7}\ \mathrm{m}^3$ (approximately the size of a consumer CPU die) could then be estimated to

$$\frac{6.1 \cdot 10^{-7}\ \mathrm{m}^3}{100^3\ \mathrm{nm}^3} = 6.1 \cdot 10^{14} \quad (1)$$

magnon logic devices per CPU. Depending on the type of magnonically realized device, one magnon gate can be the equivalent of multiple transistors. If instead of the die, the package size required for heat distribution to the heat sink is considered, this figure rises to 10^{16} magnon gates in a volume of 40 mm by 40 mm by 5 mm.

Taking into account the significant reduction in production costs when considering self-reproducing bacteria for manufacturing, it becomes feasible to also take the volume occupied by the heat sink and active cooling equipment into

account. However, it is unlikely that such big volumes will be considered for consumer magnonics, instead more powerful small devices are likely to be built. For the "equivalent volume magnon processor" shown in supplementary Fig. 1, the error bar reflects uncertainties about the size of the magnonic devices, the size of the particles used, the assembly mechanism and spacing between individual gates, as well as the realization of connections. Most notably, however, nanomagnonic logic gates open a whole new power law, similar to Moore's law, as they can be assembled as 3-dimensional structures right away. This becomes apparent when considering, that a change in the size of a transistor by some Δx yields an increase in the transistor count proportional to Δx^{-2} in 2-dimensional silicon technology, whereas in 3-dimensional systems a Δx^{-3} suggesting a faster rising power law.

SUPPLMENTARY FIGURES

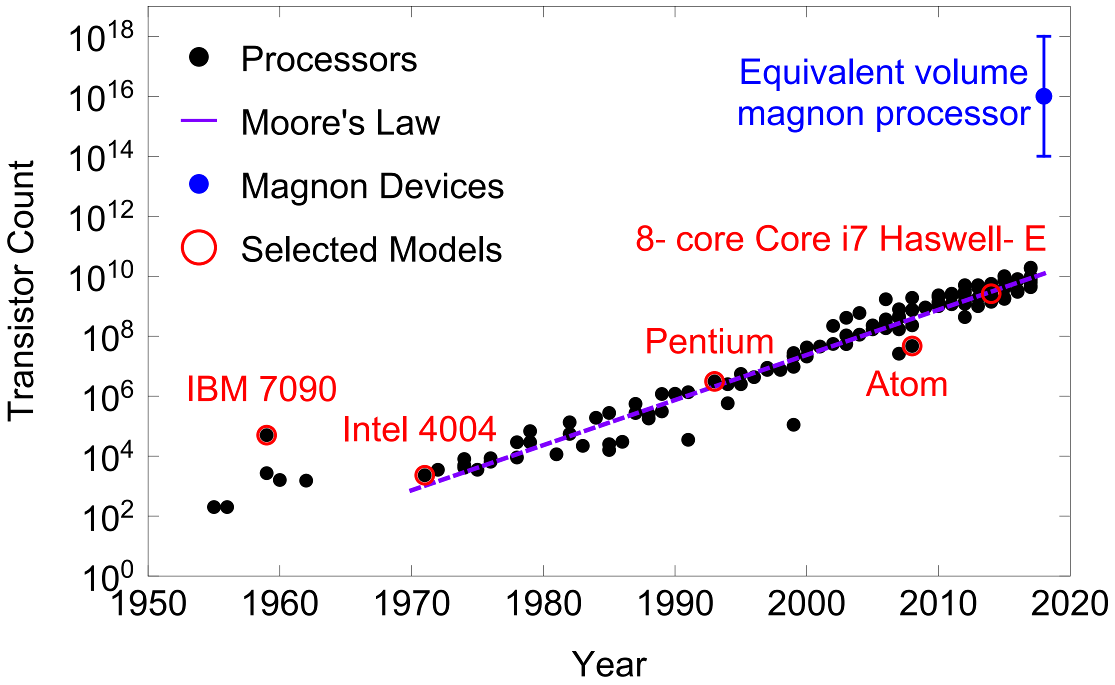

Supplementary Fig. 1 Evolution of transistor count per CPU over time. Black dots indicate various CPU Models[1]. Some Models are highlighted with a red circle. The purple dashed line indicates Moore's law: $c = 2^{(yr-1951)/2}$, which is limited by the transistor size and cooling requirements. The blue bar indicates the regime where a magnon processor would settle with the same volume as the highlighted Core i7. See supplementary text for a calculation of the effective transistor count of an equivalent magnon processor.

[1] A list can be found here: https://en.wikipedia.org/w/index.php?title=Transistor_count&oldid=856398251

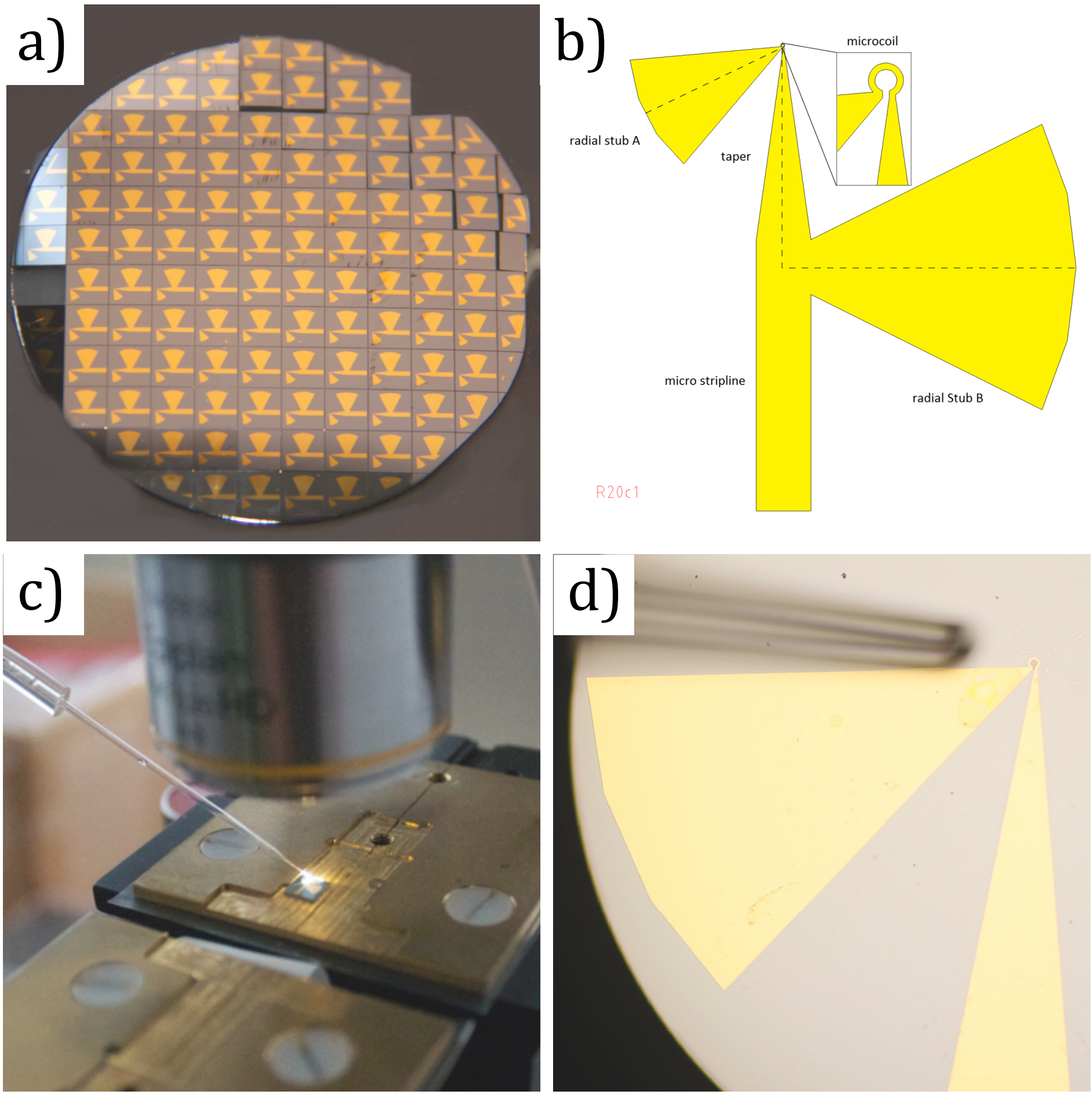

Supplementary Fig. 2: (a) Wafer with optically produced microresonators (b) Schematic view of microresonator layout with all matching stubs designed to accommodate a standing wave (dashed) at ca. 9 GHz. The sample is positioned in the omega-shaped microcoil (inset, ca. 20 µm loop diameter) and experiences an RF magnetic field produced by the microwave electric current. The RF magnetic field at the sample position is perpendicular to the substrate place. (c) loading of microresonator under an upright microscope with capillary mounted on micromanipulator. (d) Close-up of capillary tip positioned above micromanipulator.

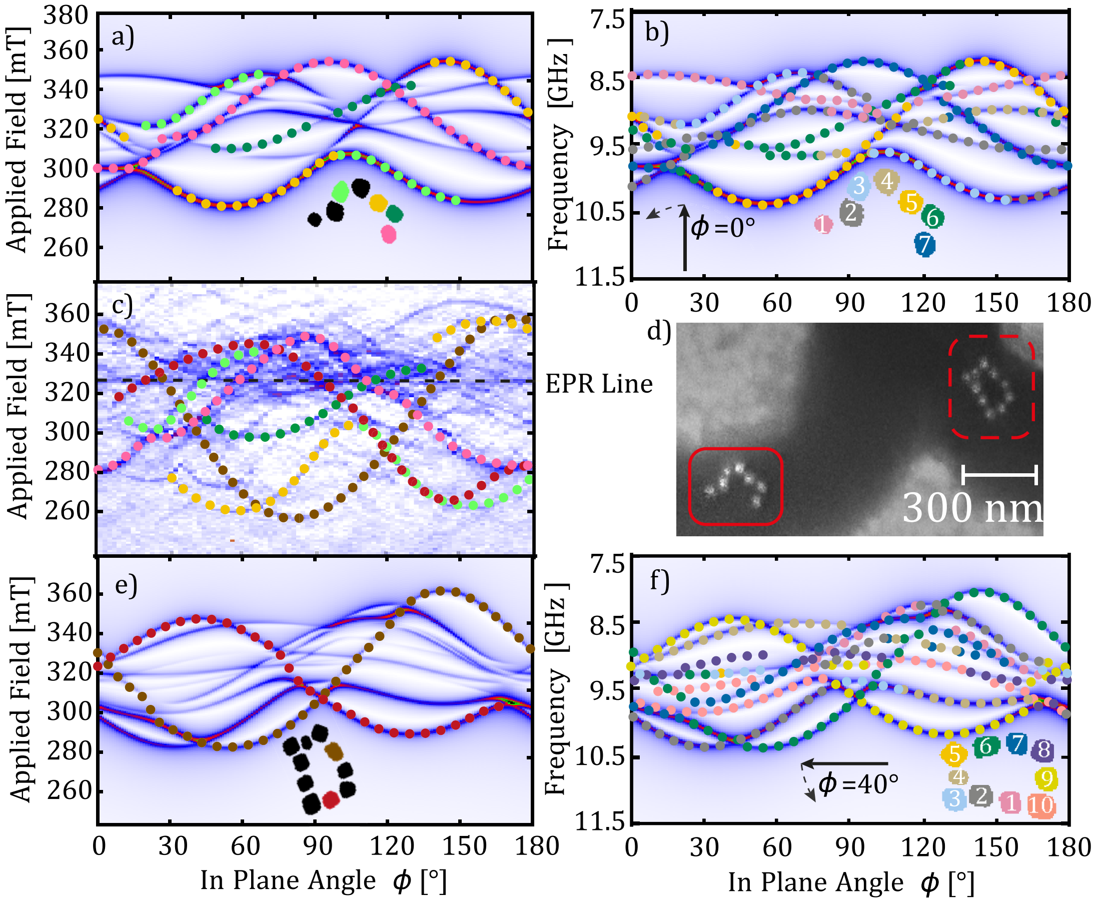

Supplementary Fig. 3: Experimental and simulated FMR spectra of 7 + 10 magnetic nanocrystals in crooked and ring-like arrangements as seen in two cells of the Δ*mamK*-mutant of MSR-1. Interrupted resonance lines (band gaps) reveal repulsive modes, while merging lines indicate attractive modes (**a, e**) Simulated FMR spectra for the curved chain (a) and the closed-loop chain (e) from electron micrograph (d). Frequency was converted to magnetic field according to Eq. (1). Lines that can be observed in the experimental specrum c are colored accordingly in a, e. (**b, f**) Majority criterion applied to a and e. The arrows indicate the direction of the applied magnetic field (at 0° in B and 40° in F relative to the orientation of the inset) and its sense of rotation (counterclockwise) relative to the chain. A description of **b)** can be found in the caption to Fig. 4a). In the closed-loop structure (e), two major uniaxial dependencies exist, offset by 90 degrees, which track the long and short axis of the structure, respectively (compare green dots vs yellow dots in f). Again, the most pronounced resonance lines represent mergers of attractive modes. (**c**) Experimental angular-resolved FMR spectra at a fixed frequency of about 9.5 GHz, recorded as in Fig. 2a; (**d**) electron micrograph of the sample containing two cells of the Δ*mamK*-mutant of MSR-1, each with coiled particle configuration.

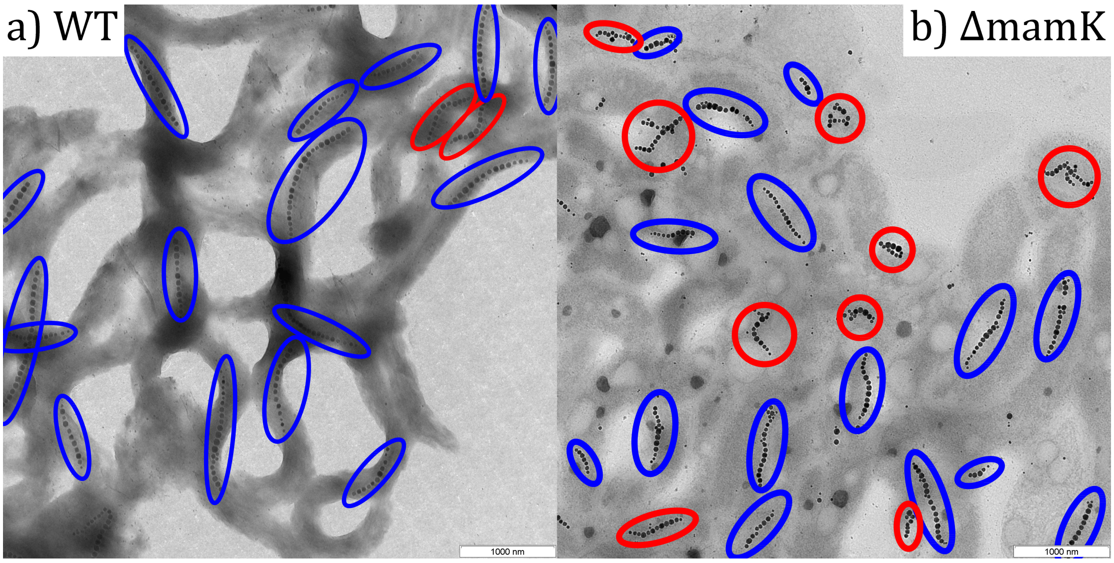

Supplementary Fig. 4: Bright-field transmission electron micrograph of sample of the wildtype (WT, a) and Δ*mamK* mutant (b) of *M. gryphiswaldense*, showing that magnetosome chains with kinks (red circles) occur significantly more often in the mutant. Red and blue circles, respectively, indicate chains with and without promising device properties.

SUPPLEMENTARY NOTE 2:

Mumax-Input File for simulation of response of 8 particle chain to an RF pulse

```
Nx := 16
Ny := 128
Nz := 9

Setgridsize(Nx, Ny, Nz)
Setcellsize(5e-9, 5e-9, 5e-9)

EdgeSmooth = 4

// Material: Fe3O4
Msat = 4.8e5 //A/m
Aex = 1.32e-11  //J/m
Kc1 = -1.10e4 //J/m^3
alpha = 0.002
gf := 2.1
muB := 9.27400968e-24
hq := 1.054571726e-34
gammaLL = gf*muB/hq

//Geometry [m]
particle1 := Ellipsoid(30.0416e-9, 30.0416e-9, 30.0416e-9).Transl(-
10.e-9, 255.e-9, 0)
particle2 := Ellipsoid(31.7014e-9, 31.7014e-9, 31.7014e-9).Transl(-
3.07143e-9, 206.929e-9, 0)
particle3 := Ellipsoid(30.7653e-9, 30.7653e-9, 30.7653e-9).Transl(-
2.95455e-9, 165.985e-9, 0)
particle4 := Ellipsoid(31.629e-9, 31.629e-9, 31.629e-9).Transl(-
10.3571e-9, 121.357e-9, 0)
particle5 := Ellipsoid(31.629e-9, 31.629e-9, 31.629e-9).Transl(-
10.3571e-9, 63.6429e-9, 0)
particle6 := Ellipsoid(32.4612e-9, 32.4612e-9, 32.4612e-9).Transl(-
7.5e-9, 12.5e-9, 0)
particle7 := Ellipsoid(30.0416e-9, 30.0416e-9, 30.0416e-
9).Transl(0.e-9, -30.e-9, 0)
particle8 := Ellipsoid(31.7429e-9, 31.7429e-9, 31.7429e-
9).Transl(1.78571e-9, -81.0714e-9, 0)

DefRegion(1, particle1)
DefRegion(2, particle2)
DefRegion(3, particle3)
DefRegion(4, particle4)
DefRegion(5, particle5)
DefRegion(6, particle6)
DefRegion(7, particle7)
DefRegion(8, particle8)

anisC1.SetRegion(1, vector(-0.48064, 0.723205, 0.495944))
anisC1.SetRegion(2, vector(0.600929, 0.790392, 0.119014))
anisC1.SetRegion(3, vector(0.533703, -0.787153, -0.309114))
anisC1.SetRegion(4, vector(0.249272, -0.835063, -0.490442))
anisC1.SetRegion(5, vector(-0.21074, 0.777473, -0.592557))
anisC1.SetRegion(6, vector(-0.744541, 0.662991, 0.0781123))
anisC1.SetRegion(7, vector(0.364565, -0.730046, -0.578036))
anisC1.SetRegion(8, vector(0.104132, -0.785258, -0.61035))
```

```
anisC2.SetRegion(1, vector(-0.0384542, 0.547627, -0.835838))
anisC2.SetRegion(2, vector(-0.794548, 0.574481, 0.196632))
anisC2.SetRegion(3, vector(-0.463721, -0.578073, 0.671412))
anisC2.SetRegion(4, vector(-0.424547, -0.549398, 0.719667))
anisC2.SetRegion(5, vector(0.544187, 0.596865, 0.589587))
anisC2.SetRegion(6, vector(0.508831, 0.63934, -0.576485))
anisC2.SetRegion(7, vector(0.297113, -0.497114, 0.815231))
anisC2.SetRegion(8, vector(0.301939, -0.559761, 0.771687))


setgeom(particle1.add(particle2).add(particle3).add(particle4).add(pa
rticle5).add(particle6).add(particle7).add(particle8))


m = RandomMag()

// applied field loop parameters
theta := 8*(pi/180) // field angle
bmax  := 0.36// max field for loop

//driving field
driv := 0.01 // amplitude driving field
f    := 1.0e9 // frequency units
fdel := 17.*f*2.*pi  // defines frequency window
time := 6400./fdel
toff := time/2.0 // offset to start after settles

outputformat = OVF1_TEXT

tableadd(B_ext)

B_ext=vector(bmax*cos(theta), bmax*sin(theta), 0)

relax() //Static Relaxation
minimize()
run(1e-12) //Dynamic Relaxation
autosave(m,pi/(2*fdel))
tableautosave(pi/(2*fdel))

B_ext=vector(bmax*cos(theta), bmax*sin(theta), driv*sin((t-
toff)*fdel)/((t-toff)*(fdel)))

run(time)
```
