# Supplementary material for: Biologically encoded magnonics
Source: Nat Commun. 2019 Sep 25;10:4345. doi: 10.1038/s41467-019-12219-0 (PMC6761176; doi:10.1038/s41467-019-12219-0)
Supplement: Supplementary file 2 — Peer Review [file 41467_2019_12219_MOESM2_ESM.pdf]

Editorial Note: This manuscript has been previously reviewed at another journal that is not operating a transparent peer review scheme. This document only contains reviewer comments and rebuttal letters for versions considered at ***Nature Communications.***

Reviewers' comments:

Reviewer #1 (Remarks to the Author):

The Authors have fully addressed my comments and, in general, did a great job with the improvement of the manuscript in the process of the reviewing. I recommend the manuscript for publication in its current form.

Reviewer #2 (Remarks to the Author):

The revised paper appears to present the results in a more accurate form with key clarifications in appropriate places. The main accomplishment is a difficult and sensitive measurement that will interest a number of groups working on magnetic particle assemblies and may inspire future work using this technique. I believe that the manuscript is suitable for publication in Nature Communications.

Reviewer #5 (Remarks to the Author):

This improved version addresses most of the referee questions.
A TEM micrograph has been incorporated in the supplementary material section. It is a image of very poor quality. It is very hard to appreciate the chain kinks. It should not be difficult to obtain a higher magnification image showing for readers the details that authors state. May be that STEM-HAADF mode should be better than TEM to improve resolution. The quality and sophisticated degree of the work merit an impressive micrograph. The readers will appreciate it.
I don't know what was the preparation of the sample for TEM. The authors do not describe in the Methods section the details of the preparation: use of contrast, embebed in resin?
The micrographs of figures 3 and 4 are really SEM images? The method contains no information.
The authors should pay attention to these issues.

Response to Reviewers:

Reviewer #1 (Remarks to the Author):

The Authors have fully addressed my comments and, in general, did a great job with the improvement of the manuscript in the process of the reviewing. I recommend the manuscript for publication in its current form.

Response: We would like to thank the referee for their positive evaluation.

Reviewer #2 (Remarks to the Author):

The revised paper appears to present the results in a more accurate form with key clarifications in appropriate places. The main accomplishment is a difficult and sensitive measurement that will interest a number of groups working on magnetic particle assemblies and may inspire future work using this technique. I believe that the manuscript is suitable for publication in Nature Communications.

Response: We would like to thank the referee for their positive evaluation.

Reviewer #5 (Remarks to the Author):

This improved version addresses most of the referee questions. A TEM micrograph has been incorporated in the supplementary material section. It is a image of very poor quality. It is very hard to appreciate the chain kinks. It should not be difficult to obtain a higher magnification image showing for readers the details that authors state. May be that STEM-HAADF mode should be better than TEM to improve resolution. The quality and sophisticated degree of the work merit an impressive micrograph. The readers will appreciate it. I don't know what was the preparation of the sample for TEM. The authors do not describe in the Methods section the details of the preparation: use of contrast, embedded in resin?
The micrographs of figures 3 and 4 are really SEM images? The method contains no information.
The authors should pay attention to these issues.

Response: We would like to thank the referee for the appraisal of our work and for calling our attention to the EM issues. We apologize for the poor pixel resolution of the TEM picture from the previous version, which most likely was caused by the conversion from word to PDF, please see the difference below on the next page:

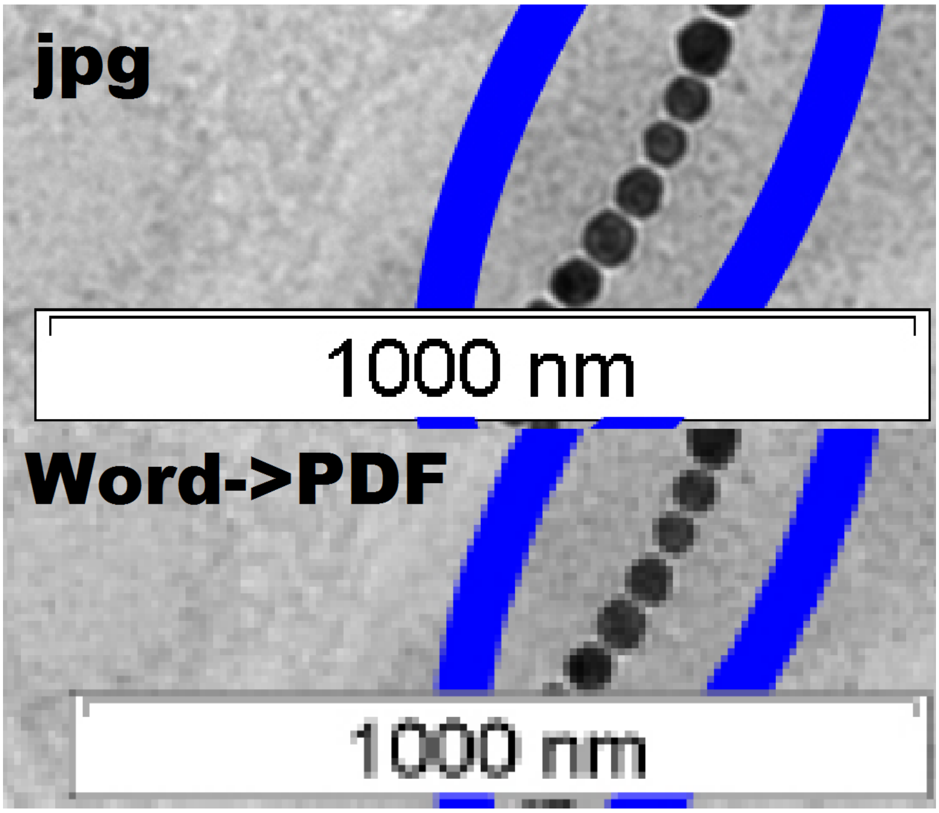

We have uploaded the original JPEG file of the mutant cells in the manuscript system.

We have clarified in the *Methods* section that the TEM micrographs were taken at 120 keV in normal bright field mode with a Zeiss 912 EM Omega.

We have clarified in the *Methods* section that the SEM micrographs shown in Fig. 2A (inset), 3E, and 4ii were all acquired with the FIB/SEM device we used to prepare the single cell sample and that they were imaged at 10 keV acceleration voltage with the secondary electron detector.

REVIEWERS' COMMENTS:

Reviewer #5 (Remarks to the Author):

The authors have addressed my comments. I believe that the manuscript is suitable for publication in Nat Comm in its current form
